# CRFVoter: gene and protein related object recognition using a conglomerate of CRF-based tools

**DOI:** 10.1186/s13321-019-0343-x

**Published:** 2019-03-14

**Authors:** Wahed Hemati, Alexander Mehler

**Affiliations:** Text Technology Lab, Goethe-University Frankfurt, Robert-Mayer-Straße 10, 60325 Frankfurt am Main, Germany

**Keywords:** BioCreative V.5, Biomedical named entity recognition, GPRO, BioNLP, Named entity recognition, CRF, Machine learning

## Abstract

**Background:**

Gene and protein related objects are an important class of entities in biomedical research, whose identification and extraction from scientific articles is attracting increasing interest. In this work, we describe an approach to the BioCreative V.5 challenge regarding the recognition and classification of gene and protein related objects. For this purpose, we transform the task as posed by BioCreative V.5 into a sequence labeling problem. We present a series of sequence labeling systems that we used and adapted in our experiments for solving this task. Our experiments show how to optimize the hyperparameters of the classifiers involved. To this end, we utilize various algorithms for hyperparameter optimization. Finally, we present CRFVoter, a two-stage application of Conditional Random Field (CRF) that integrates the optimized sequence labelers from our study into one ensemble classifier.

**Results:**

We analyze the impact of hyperparameter optimization regarding named entity recognition in biomedical research and show that this optimization results in a performance increase of up to 60%. In our evaluation, our ensemble classifier based on multiple sequence labelers, called CRFVoter, outperforms each individual extractor’s performance. For the blinded test set provided by the BioCreative organizers, CRFVoter achieves an F-score of 75%, a recall of 71% and a precision of 80%. For the GPRO type 1 evaluation, CRFVoter achieves an F-Score of 73%, a recall of 70% and achieved the best precision (77%) among all task participants.

**Conclusion:**

CRFVoter is effective when multiple sequence labeling systems are to be used and performs better then the individual systems collected by it.

## Introduction

The research fields of biology, chemistry and biomedicine have attracted increasing interest due to their social and scientific importance and also because of the challenges arising from the intrinsic complexity of these domains. Like many other research areas, they are currently changing due to the rapid development of machine learning (ML) and artificial intelligence (AI). ML is used in many of these research areas. For instance, in the biomedical area it is used for biomedical signal processing (BSP) [1, 2], biomedical imaging (BI) [3–5] and disease prediction through patient profiling [6]. The former approaches work with structured data such as EEG data in the case of BSP. The last two approaches work with unstructured data such as MRI for BI and doctor-patient conversations in the case of disease classification and differential diagnosis [7–10]. The growth in the amount of publicly available data has led to enormous efforts to develop, analyze and apply new learning methods in the field of chemistry and biology. This concerns, for example, virtual screening [11] for drug design and drug discovery [12, 13]. In order to advance areas of biological, chemical and biomedical research, it is important to perform state-of-the-art algorithms of data analysis. In carrying out scientific work, most researchers rely on published information to keep abreast of the latest developments in these fields, to avoid repetition and determine the direction of current studies. Numerous new publications appear daily in biomedical journals, in the form of scientific articles, patent applications, reports from health authorities and other text collections on the Internet, making it difficult to keep pace with the development of this discipline. Thus, there is an increasing interest in improving access to information on biological, chemical and biomedical data described in such texts and text repositories. To achieve this goal, a fundamental step is to automatically identify biological and chemical entities in these repositories. Based on this identification, interactions between drugs and proteins, for example, can be detected, side effects of chemical compounds and their associations to toxicological endpoints can be identified or information about metabolic reactions can be extracted [14].

For these reasons, initiatives and call for participation in corresponding competitions have been launched in recent years by professional communities that describe challenges in the identification of biochemical units. One of these initiatives is the BioCreative series which focuses on biomedical text mining. BioCreative is a “Challenge Evaluation”, in which the participants are given defined text mining or information extraction tasks in the field of biology. These tasks include Gene Mention detection (GM) [15, 16], Gene Normalization (GN) [15, 17, 18], Protein–Protein Interaction (PPI) [19], Chemical Compound and Drug Name Recognition (CHEMDNER) [20] and Chemical Disease Relation Extraction (CDRE) [21, 22] tasks.

The current *BioCreative V.5* task consists of two off-line tasks, namely *Chemical Entity Mention in Patents (CEMP)* and *Gene and Protein Related Object Recognition (GPRO)*. CEMP requires the detection of chemical named entity mentions. The task requires detecting the start and end indices corresponding to chemical entities. The GPRO task requires identifying mentions of gene and protein related objects mentioned in patent titles and abstracts [23]. In this work, we focus on the second task, that is, the GPRO task. The GPRO task is an abstraction of the well-known Named Entity Recognition (NER) tasks, which can be reduced to a sequence labeling problem, where input sentences are represented as sequences of tokens. The task is then to tag genes and protein-related mentions in these sequences of sentences. The present paper addresses this task and is an extension of previous work [24].

The paper is organized as follows: In "Methods" section we describe our methodical apparatus and resources. First, we describe the data used for this work. We then present state-of-the-art tools for NER and how we adapted them for applying them in the biological domain. We examine the impact of hyperparameter optimization and show that it brings a considerable boost in performance. Next, we present a novel tool, called CRFVoter, for combining sequence labeling tools as used in our hyperparameter optimization. In "Results" section, we present and discuss our results and in "Conclusion " section we conclude and shed light on further work.

## Methods

### Dataset

The organizers of *BioCreative V.5* provided a corpus of 30 000 patent abstracts (titles and abstracts in English) from patents published between 2005 and 2014, where 21 000 of them are used as a training set and the remaining 9 000 as a test set. The corpus is manually annotated for the GPRO tasks. Gene and protein related object annotations were divided into type 1 and type 2. Type 1 are those GPRO mentions that can be normalized to database entries, like UniProt^1^, NCBI^2^, OMIM^3^, GeneCards^4^, FlyBase^5^, etc. Type 2 are those mentions that cannot be normalized. Table 1 shows the number of instances of type 1 and type 2 annotations in the GPRO Task. 5795 documents from the 21,000 documents of the training set contained GPRO mentions. To reduce noise during training, only the annotated subset of 5795 documents were considered; from now on, the collection of the documents will be called *filtered corpus*. Then, by means of random sampling, the filtered corpus was divided into three sets: 60 % of the document were sampled into the training set, 20 % into the development set and 20 % into the test set. The filtered corpus had been enriched with additional linguistic features. To this end, multiple preprocessing steps were applied on each of the three sets including sentence splitting, tokenization, lemmatization, part-of-speech tagging and fine-grained morphological tagging by means of the Stanford CoreNLP [25] and TextImager [26]. In addition, tokens were split on non-alphanumeric characters, as this variant brought a performance increase. Table 2 lists the number of documents, sentences and tokens of the filtered corpus. Since the GPRO task can be reduced to a sequence labeling problem, the filtered corpus was converted into a sequence structure. To this end, a sequence of documents each containing a sequence of sentences each containing a sequence of tokens was constructed. This results in a file in TSV format, where each word and its associated features are in one line separated by tabs. Sentences are separated by an empty line. For the labeling of the GPRO mentions, the IOB tagging scheme [27] was used (*I* = inside of a entity, *O* = outside of a entity, *B* = beginning of a entity). This approach allows for the annotation of entities that span multiple tokens. Note that the beginning and end of each entity mention is marked. This allows models to not only learn tags themselves, but also the corresponding transition probability. Between all beginning and end tags, the inside parts, for example, should also be part of the manifestation of the entity. It is worth noticing that using the IOB scheme has also disadvantages. The smallest unit that can be annotated is a token. Consider, for example, the token “**B-Raf**V600E”. Only “**B-Raf**” is annotated in the gold standard. This cannot be represented using the IOB format. To solve this problem, a tokenizer has to be developed that covers exactly these special cases. The filtered corpus contains 0,85% of these special cases. Since their recognition cannot be trained, they have been removed from the training set. However, during evaluation, these cases were considered as errors. In all experiments described in the following sections, we used the corpus as described so far.

**Table 1:** Number of instances of type 1 and type 2 in GPRO task

Type 1	Number	Type 2	Number
ABBREVIATION	7516	ABBREVIATION	27
FAMILY	1	FAMILY	5029
FULL NAME	4815	FULL NAME	27
IDENTIFIER	1	MULTIPLE	178
NESTED	89	NO CLASS	45
		SEQUENCE	23
Total count:	12,422	Total count	5329

**Table 2:** The number of documents, sentences and tokens of the filtered corpus

# Documents	5795
# Sentences	19,673
# Tokens	633,928

### System description

In this section we describe CRFVoter. Our approach implements a two-stage application of Conditional Random Fields (CRF) [28] using a conglomerate of sequence labelers for the detection of mentions of gene and protein related objects in biomedical patent abstracts. We trained and optimized five NER for tackling the GPRO task. We also optimized the hyperparameter settings of each of these NERs. Hyperparameter tuning is a challenging task in ML in the sense that the optimal set of hyperparameters depends on the model, the dataset and the domain [29] forming a huge interactive parameter space. In this context, our experiments focused on optimizing the hyperparameters of each NER system independently. This led to a noticeable increase of F-score compared to the default settings. For each NER, we performed a hyperparameter optimization by means of the *Tree-structured Parzen Estimator (TPE)* [30]. The NERs are more or less independent of each other in the sense that one can always find a subset of test cases being processed correctly by one NER but not by any other one. Therefore, combining these NERs is a promising candidate for increasing precision and recall. We started with computing combinations of these NERs by means of a simple majority vote [31]. Majority voting means to select the target label that is assigned by the majority of classifiers. Our experiments show that a simple majority vote brings no gain in precision and recall compared to the best performing reference systems being examined in our study. Thus, we alternatively experimented with a two-stage model, called CRFVoter, which trains a CRF to learn the best combination of the underlying sequence labeling tools (i.e. our case these are the NERs). We show, that CRFVoter outperforms every reference systems being examined in our study. In the rest of this section, we present a survey of hyperparameter optimization algorithms and discuss why TPE is the best optimization algorithm for our studies. We present a survey of NERs trained for the GPRO tasks and the parameter settings optimized by means of the TPE hyperparameter optimization algorithm. This includes the NER systems described in the following subsections. Finally we describe the ensemble classifiers based on majority voting and on our CRFVoter.

#### Hyperparameter optimization

In this section, we describe the concepts of hyperparameter tuning. A ML model consists of various parameters that must be learned using the underlying training data. The main task of ML is to adapt a model to the given data. This process of fitting the model parameters to existing data is called *model training*. Hyperparameters are a class of parameters that cannot be learned directly from the training process. The hyperparameters are the variables that govern the training process itself. These parameters must be predefined; they define higher-level concepts about the model, such as complexity, convergence rate, penalty, and so on [30]. Hyperparameters are configuration variables of the training process that are normally kept constant. Hyperparameter optimization, also called hyperparameter tuning, is used to find optimal hyperparameter configurations for a ML algorithm on a given dataset. The goal is, to find optimized values for hyperparameters, which maximize the prediction accuracy of a model. Hyperparameter tuning works by performing several trials of the same training job. Each trial is a complete execution of the training process with values for pre-selected hyperparameters that are within predefined limits. Hyperparameter tuning optimizes one or more target variable where this variable is also called performance metric or hyperparameter metric [32]. In our case we have considered a single target variable, that is, the F-score, because this is usually or at least predominantly done in NER. The hyperparameters are adjusted by running the entire training job, so that overall hyperparameter metric is improved. Since parameter spaces tend to include more and more dimensions, it is usually not possible to search the entire space to find the optimal configuration. Therefore, approximation algorithms must be used to maximize the hyperparameter metric (locally or globally). In the next sections we introduce a general notation and describe some hyperparameter optimization algorithms.

*General notation* Following the notation of [32, 33], a ML algorithm $\mathcal {A}$ is a mapping $\mathcal {A}: \mathcal {D} \rightarrow \mathcal {M}$ where $\mathcal {D}$ is the dataset and $\mathcal {M}$ is the space of all models. $\mathcal {A}$ has *n* hyperparameters, denoted as $\theta _1,\ldots ,\theta _n$ and a configuration space $\Theta = \Theta _1 \times \ldots \times \Theta _n$ with $\theta _i \in \Theta _i, i = 1,\ldots ,n$. The learning algorithm estimates a model $M(\varvec{\theta }) \in \mathcal {M}$ that minimizes a loss function $\mathcal {L}$, given a hyperparameter configuration $\varvec{\theta }=\langle \theta _1,\ldots ,\theta _n \rangle$ on the training data $\mathcal {D}^{(train)}$:

1 $$\mathcal {A}_{\varvec{\theta }}(\mathcal {D}^{(train)}) := \underset{M(\varvec{\theta }) \in \mathcal {M}}{\arg \min } \mathcal {L}(M(\varvec{\theta }),\mathcal {D}^{(train)})$$

The goal of hyperparameter optimization is then to find the optimal configuration $\varvec{\theta }^*$ using a validation set:

2 $$\varvec{\theta }^*:=\underset{\varvec{\theta }\in \Theta }{\arg \min } \mathcal {L}(\mathcal {\mathcal {A}_{\varvec{\theta }}}(\mathcal {D}^{(train)}),\mathcal {D}^{(valid)})$$

*Grid Search* Grid Search is a widely used hyperparameter optimization algorithm. It searches through a manually specified subset $\Theta _U \subset \Theta$ of the hyperparameter space. In a grid search, the set of trials is formed by assembling every possible configuration $\varvec{\theta }$ of values in $\Theta _U$, so the number of trials in a Grid Search is $|\Theta _U|$ elements [34]. For each hyperparameter configuration $\varvec{\theta }\in \Theta _U$ a model $M(\varvec{\theta })$ is estimated and tested against the validation set $\mathcal {D}^{(valid)}$. This makes Grid Search suffering from the *curse of dimensionality* [35] because the number of joint values in $\Theta _U$ grows exponentially with the number of hyperparameters. Since Grid Search works on a grid, continuous parameters must be discretized. In our experiments we used Grid Search in cases in which $|\Theta |<200$ and where the parameter space did not contain continuous parameters—under these conditions, Grid Search will find the optimal configuration in foreseeable time.

*Random Search* Random Search is an optimization algorithm that searches a hyperparameter space $\Theta$ by selecting random hyperparameter configurations. Unlike Grid Search, no subset $\Theta _U \subset \Theta$ of the hyperparameter space must be defined. Instead, the parameters of a setting $\varvec{\theta }\in \Theta$ are randomly selected. The advantage of this approach is that not only discrete parameters can be selected, but also continuous and mixed parameter spaces. Bergstra et al. [34] found, that randomly chosen trials are more efficient for hyperparameter optimization then trials on a grid. They show empirically and theoretically that random searches are more effective for parameter optimization than grid searches when considering the same number of trials.

*Bayesian Optimization* Bayesian Optimization is a model-based optimization process for black box functions. The Bayesian optimization searches for the maximum of an unknown target function. It employs the Bayesian technique of setting a prior over the objective function and combining it with evidence to get a posterior function. Bayesian Optimization uses a Gaussian process [36] to model the surrogate. It optimizes the expected probability that new trials will improve compared to the best current observation. The Gaussian process is a distribution over functions, which involves adapting this distribution to the given data, so that functions are generated that come close to the observed data. This distribution is further optimized by iteratively selecting the next point, which must take into account both exploration (sampling from areas of high uncertainty) and exploitation (sampling areas likely to offer improvement over the current best observation) [37]. Applied to hyperparameter optimization, Bayesian optimization builds a probabilistic model that assigns the hyperparameter values to the hyperparameter metric evaluated on the validation set. It has been shown that Bayesian optimization achieves better results in fewer trials than Grid Search and Random Search [38].

*Tree-structured Parzen Estimator* The Tree-structured Parzen Estimator [30] is a sequential model-based optimization (SMBO) [39] approach. SMBO methods sequentially construct models to approximate the performance of hyperparameters based on “historical” (that is, preceding) measurements. For each iteration, TPE collects new observation, where at the end the algorithm decides which set of parameters it should try next. The main idea is similar to Bayesian Optimization (see "Hyperparameter optimization" section). However, it fixes disadvantages of the Gaussian Process used by Bayesian Optimization. The TPE approach models *P*(*x*|*y*) and *P*(*y*) where *x* represents hyperparameters and *y* the associated hyperparameter metric. *P*(*x*|*y*) is modeled by transforming the generative process of hyperparameters, replacing the distributions of the configuration prior with non-parametric densities. For the first few iterations TPE performs a Random Search. The next step is to divide the collected observations into two groups. The first group contains observations that yielded the best results after the evaluation and the second group contains the remaining observations. The goal is to find a set of parameters that are more likely to be in the first group and less likely to be in the second group. In contrast to Bayesian Optimization, TPE no longer relies on the best observation. Instead, a distribution over the best observations is used. The next step of the TPE is to model the likelihood probabilities for each of the two groups. This is the next big difference to the Gaussian Process. Gaussian Process models posterior probability instead of likelihood probability. Candidates are sampled using the likelihood probability from the group containing best observations. From the sampled candidates TPE tries to find a candidate that is more likely in the first group *l*(*x*) and less likely in the second group *g*(*x*); this is done by means of the *Expected Improvement* (EI):

3 $$EI(x)=\frac{l(x)}{g(x)}$$

From the sampled candidates, the parameter setting that has the highest Expected Improvement is selected for the next iteration. The optimization process ends after a predefined number of iterations.

#### Sequence labeling systems

In this section we describe the sequence labeling systems used in our experiments. These are state-of-the-art systems based on different architectures, namely CRF and Neural Networks. We show that hyperoptimization brings a considerable increase in performance. Finally, we present two variants for ensemble classifiers, namely Majority Voter and the CRFVoter.

**Fig. 1:**
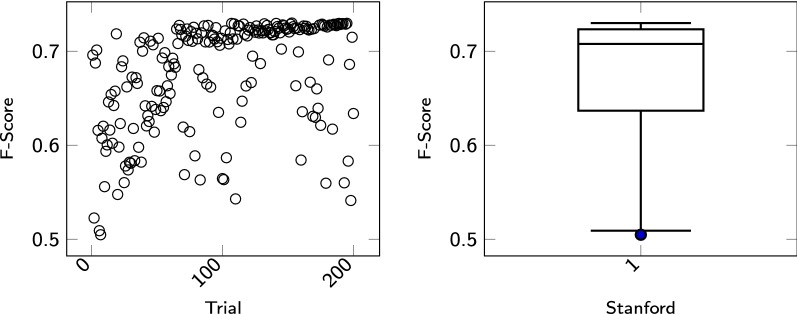
The figure shows the results of optimizing StanfordNER by means of TPE. The scatter plot on the left side shows the results of each trial. The boxplot shows in which area the results are located and how they are distributed over this area. The difference between the best and the worst performing setting is 23%

*Stanford Named Entity Recognizer* Stanford Named Entity Recognizer^6^ (StanfordNER) is a Java implementation of CRF based Named Entity Recognizer [40]. Finkel et al. [41] has participated in BioCreative to explore StanfordNER’s limitations in the biological domain. They participated in BioCreative I Task 1A [42] and achieved the best performance in the open task and the second best performance in the closed task. For StanfordNER our experiments are based on their results. The StanfordNER has since been further developed. New parameters have been added, which we have taken into account in our experiments. Table 3 shows the corresponding hyperparameter space used in our experiments. Since the parameter space is so large that one cannot search it with a grid search, a hyperparameter optimization algorithm must be used. For our experiments we optimized the hyperparameters by means of TPE (see "Hyperparameter optimization" section). During the optimization process we ran 200 trials to approximate the optimal parameter setting. The results of the trials are plotted in Fig.  1 in the scatter plot. The scatter plot shows that the F-score converges towards 73%. On the right side of Table 1 one sees the graphical representation of the F-Score distribution using a boxplot. The significance of a parameter study becomes immediately clear in this example. Depending on the parameter setting, the results vary by 23%. The best performing set of features forGPRO, marked with italic font, leads to an F-score of 0,73. The worst setting results in an F-score of 0,50.

**Table 3:** Parameter space of stanford named entity recognizer used in our experiments. The column *Possible values* describe the range of the parameters. The parameter setting with the best value is highlighted in italic

Parameter	Possible values
useClassFeature	[*true*,false]
useWord	[*true*,false]
useNGrams	[*true*,false]
noMidNGrams	[true,*false*]
normalizeTerms	[true,*false*]
usePosition	[*true*,false]
useNeighborNGrams	[true,*false*]
useMoreNeighborNGrams	[*true*,false]
usePrev	[true,*false*]
useNext	[*true*,false]
useTags	[*true*,false]
useWordPairs	[*true*,false]
useDisjunctive	[true,*false*]
useSequences	[*true*,false]
usePrevSequences	[*true*,false]
useNextSequences	[true,*false*]
useLongSequences	[*true*,false]
useTaggySequences	[*true*,false]
useSymWordPairs	[true,*false*]
useSymTags	[*true*,false]
useTypeSeqs	[*true*,false]
useTypeSeqs2	[*true*,false]
useTypeySequences	[*true*,false]
wordShape	*chris2useLC*
maxLeft	[1,*2*,3,4,5,6]
maxRight	[1,*2*,3,4,5,6]
maxNGramLeng	[1,2,3,*4*,5,6]
sloppyGazette	[true,*false*]
useGazFeatures	[*true*,false]
useWordTag	[*true*,false]
useWideDisjunctive	[*true*,false]
useLemmas	[*true*,false]
usePrevNextLemmas	[*true*,false]

**Fig. 2:**
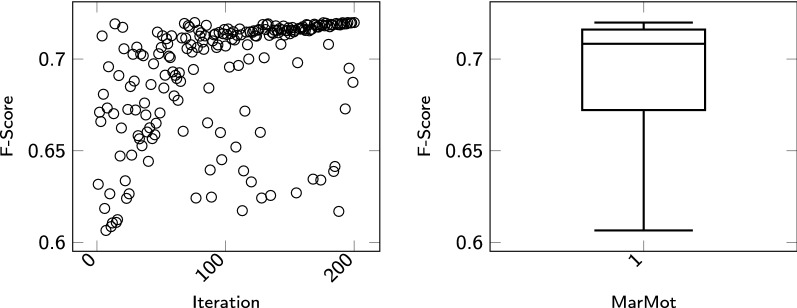
The scatter plot on the left side of the figure shows the results of the optimization process of MarMoT. The boxplot shows in which area the results are located and how they are distributed over this area. Between the best and the worst setting are 11%

*MarMoT* MarMoT^7^ is a generic CRF framework [43]. It implements a higher order CRF with approximations such that it can deal with large output spaces. Additionally it can be trained to fire on the predictions of lexical resources (so-called gazette files) and on word embeddings [43–47]. Table 4 shows the hyperparameter space used in our experiments for MarMoT. We ran 200 trials. The results of the iterations are shown in Fig. 2 using a scatterplot. One can see that the F-score converges towards 0,72. The right side of Fig. 2 shows the boxplot of the corresponding F-Score distribution. The best performing set of features for GPRO produces an F-score of 0,72. The worst set results in an F-score of 0,59. Once more, this difference hints at the importance of hyperparameter optimization.

**Table 4:** Parameter Space of MarMoT Tagger used in our experiments. The column *Possible values* describe the range of the parameters. The parameter setting with the best value is highlighted in italic

Parameter	Possible values
Num iterations	[10,*20*]
Penalty	[*0*,1,2]
Beam size	[*1*,2,5]
Quadratic penalty	[*0*,1,2]
Order	[*1*,2,3,4]
Prob threshold	[0.01,*0.001*]
Effective order	[*1*,2,3]
Num chunks	[*2*,5,10]

**Fig. 3:**
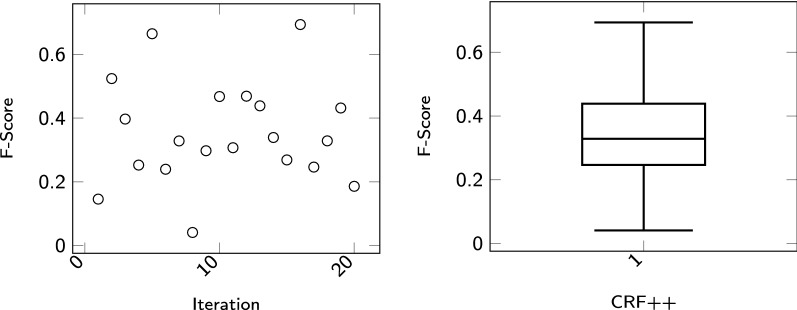
This figure shows the results of using CRF++ in conjunction with Grid Search. Due to the low dimensionality of the underlying parameter space, a Grid Search was used. The scatterplot on the left side shows the results of the optimization process for each trial. On the right side, one sees in which area the results are located and how they are distributed

*CRF++* CRF++^8^ is a customizable open source implementation of CRF [48]. In our experiments with CRF++ we used unigram and bigram features including the current, the previous and the next word. Table 5 shows the hyperparameter space used in our experiments for CRF++. The combination of parameters results in 20 model files, which is small enough to search the entire parameter space with Grid Search. The results are shown in Fig. 3. The best performing set of parameters for GPRO generates an F-score of 0,69. The worst one results in an F-score of 0,04.

**Table 5:** Parameter Space of CRF++ used in our experiments. The column *Possible Values* describe the range of the parameters. The parameter setting with the best value is highlighted in italic

Parameter	Possible values
c	[0.6, 1, 1.6, 3, 5, 7, *15*, 50, 100, 1000]
a	[CRF-L1, *CRF-L2*]

*MITIE* MITIE is an open source information extraction tool. MITIE can be trained using techniques like distributional word embeddings [44–47] and *Structural Support Vector Machines* [49]. Due to the lack of documentation, we did not optimize MITIE. The default configuration for named entity recognition produces an F-score of 0,65 for GPRO.

*Glample NER Tagger* Glample NER Tagger is a neural-network-based named entity recognizer. It is based on Bidirectional LSTMs and CRFs [50]. Due to the long-lasting training time, only the default parameter settings were considered. This resulted in an F-score of 0,74 for GPRO.

*Majority Vote* By means of majority voting, we combined the best performing outputs of each of the NER systems considered so far. We selected the label that was most frequently output by the different NER systems. Majority voting reaches an F-score of 0,68 for GPRO, which is below the best performing system considered so far. Facing these results we can state that a simple majority vote brings no gain in precision and recall. Therefore, we need an alternative considered next.

*CRFVoter* CRFVoter is a two-stage application of CRF using a conglomerate of sequence labelers. In the first step, each NER $c_m, m = 1..l,$ is optimized independently on the training set, where the *i*th sequence $t_i$ of length *n* of the set of training examples is of the form

4 $$t_i = \langle (\vec {x}_1, y_1), \ldots , (\vec {x}_n, y_n)\rangle$$

**Fig. 4:**
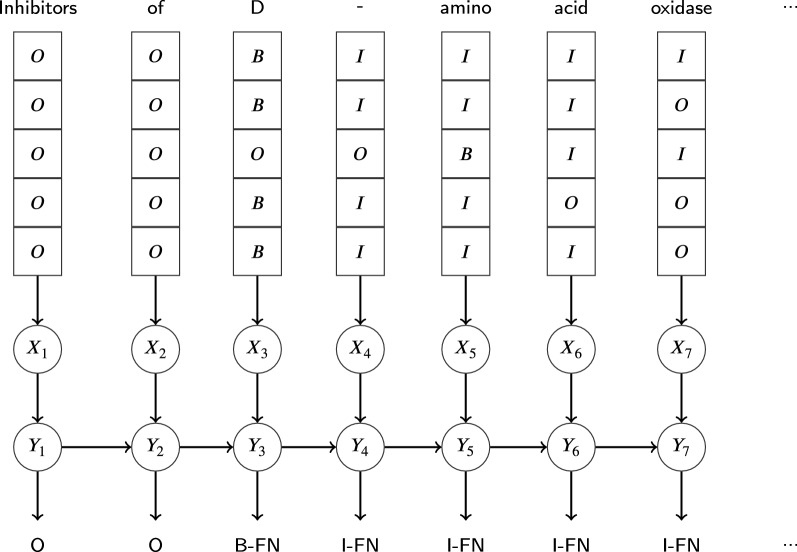
Architecture of CRFVoter exemplified by means of a single sentence

$\vec {x}_j, j = 1\ldots n,$ is a feature vector corresponding to an element in the input sequence at position *j*—in our case this corresponds to a token. $y_j$ is the corresponding discrete label of the element at position *j*—in our case this is the IOB2 formatted GPRO annotation label. The goal of a sequence labeling classifier *c* is to approximate the function $f(j)=y_j$ where $y_j$ is the true label to be assigned to the input stream at position *j*. Approximations of *f* are computed by hyperoptimizing each classifier *c* as described above. After the training phase, a development set, which is independent of the training and the test set, is tagged by means of each NER $c_m$. The output label assigned by $c_m$ is then taken by CRFVoter as an individual feature input. In the second step, CRFVoter combines each NER $c_m$ into an ensemble classifier $c = \texttt {CRFVoter}(\{c_1, c_2, \ldots , c_l\})$. The sequence of training examples used to train CRFVoter is of the form

5 $$t_i = \langle (f_{c_1}(\vec {x}_1), f_{c_2}(\vec {x}_1), \ldots , f_{c_l}(\vec {x}_1)), y_1), \ldots , ((f_{c_1}(\vec {x}_n), f_{c_2}(\vec {x}_n), \ldots , f_{c_l}(x_n)), y_n\rangle$$

where $f_{c_m}(\vec {x}_j), m=1\ldots l, j = 1\ldots n,$ is the output label of classifier $c_m$ computed for the input vector $\vec {x}_j$ at the *j*th position of the input sequence. That is, in stage one of CRFVoter, we calculate for each NER $c_m$ and each token at position *j* of the input stream a corresponding output label $f_{c_m}(\vec {x}_j)$. In the second stage, these output labels are taken as features to feed our CRF operating on the same position *j*. In this way, we train CRFVoter based on a sequence of the latter feature sets, which is exemplified in Fig. 4. Let *x* be the sequence of observed words in $t_i$ and *y* be the sequence of states that correspond to the labels assigned in $t_i$. Linear-chain CRFs define the conditional probability of a state sequence to be [28]:

6 $$P(y|x) = \frac{1}{Z_x}exp\left( \sum \limits _{j=1}^n \sum \limits _{m=1}^l \lambda _m f_m(y_{j-1},y_j,x,j)\right)$$

$Z_x$ is the normalization factor that makes the probability of all state sequences sum to one; $f_m(y_{j-1},y_j,x,j)$ is a feature function, and $\lambda _m$ is a learned weight associated with feature $f_m$. Feature functions measure the aspect of a state transition, $y_{j-1},y_j \rightarrow yt$, and the entire observation sequence, *x*, centered at the current time step, *j*. Consider, for example, Fig. 4. One feature function might have value 1 in cases where $y_{j-1}$ denotes the state B-FULLNAME, $y_j$ the state I-FULLNAME, and $X_4$ being the feature vector at position *j*. Large positive values for $\lambda _m$ indicate a preference for such an event, whereas large negative values make the event unlikely. During tagging, CRFVoter takes again the output of each NER as input features and labels the sequence by means of the 2nd level CRF.

Our experiments show that CRFVoter brings 2% gain in F1-measure compared to the best performing reference systems being examined in our study. When operating on the blinded test set for GPRO provided by the BioCreative team, CRFVoter reaches an F-score of **0,75** for the evaluation of type 1 and of type 2.

## Results

This section presents the results of our experiments for the GPRO task. For the evaluation of the GPRO Task the BioCreative Team has specified standard evaluation statistics, namely precision (P), recall (R) and F1-score (F) [51]. Three main result types were examined. *False Negative*s (FN), that is, results corresponding to incorrect negative predictions. FN are cases that were part of the gold standard but overlooked by our systems. *False Positive*s (FP) are results of false positive predictions, that is, cases predicted by our system but not so marked in the gold standard. The third type of result is *True Positive*s (TP), i.e. results consisting of annotations predicted by our system and belonging to the gold standard as such. Recall is the fraction of correctly labeled positive results and all positive cases:

7 $$R = \frac{TP}{TP + FN}$$

Precision is the fraction of all correctly labeled positive results and all labeled results:

8 $$P = \frac{TP}{TP + FP}$$

F1-score is the harmonic mean of precision and recall:

9 $$F1 = 2*\frac{P*R}{P+R}$$

In "System description" section, the results of the hyperparameter optimization are visualized. For each sequence labeling tool, the hyperparameters were optimized using TPE or, if possible, using Grid Search. The results of the trials are plotted in scatterplots and the distribution of the results are visualized in the respective boxplots. The boxplots show the big spread of the outcomes of the trials during the respective optimization processes. For example, in the optimization process of CRF++, the difference between the worst to the best performer is 60%. The results show the need for ML algorithms to perform hyperparameter optimization.

Table 6 shows the comparison of annotators trained for the GPRO task. The results listed are those obtained after the hyperparameter optimization described in "Hyperparameter optimization" section, which were trained, optimized and tested on the corpus described in "Dataset" section. Each sequence labeling system classifies a different subset correctly. Table 7 shows the pairwise differences between the sequence labeling systems. The combination of the sequence labeling systems to a Majority Voter did not bring any performance increase and is even 5% below the best performer among the sequence labeling systems. In contrast, the CRFVoter increases the performance and is the best performer in our experiments. The performance values for the official BioCreative test set were created by training each model on the entire filtered corpus (see Section "Dataset" section) and then evaluated on the official test set provided by BioCreative. For the blinded test set provided by the BioCreative organizers for GPRO, CRFVoter achieves an F-score of 75%, Recall of 71% and Precision of 80%. For the GPRO type 1 evaluation, CRFVoter achieves an F-Score of 73%, Recall of 70% and obtained the best precision (77%) achieved among all task participants.

**Table 6:** Comparison of annotators trained an tested on the filtered corpus described in "Dataset" section

System	P	R	F
Stanford NER	0,77	0,69	0,73
MarMoT	0,76	0,69	0,72
CRF++	0,75	0,64	0,69
MITIE	0,74	0,58	0,65
Glample	0,78	0,72	0,74
Majority vote	0,64	0,72	0,68
CRFVoter	0,75	0,77	0,76

**Table 7:** Differences of labeled output between each pair of NER system

	Stanford	MarMoT	CRF++	MITIE	Glample
Stanford	0	2.29 %	2.12 %	2.44 %	2.50 %
MarMoT		0	2.56 %	2.61 %	2.43 %
CRF++			0	2.91 %	2.47 %
MITIE				0	2.51 %
Glample					0

Table 6 indicates that Glample and CRFVoter might be statistically tied. To investigate the significance of the improvements we used McNemars chi-square test [52] for *labeling disagreements* between Glample and CRFVoter with $\alpha =0.05$. For both methods, we treated the predicted IOB-Tags for the test set that agreed with the gold annotations as positive, otherwise negative. For the McNemar test we only count the spans corresponding to biomedical named entities. We found that the comparison between Glample and CRFVoter is *significant* ($\rho < 0.05$) in terms of the test of [52].

## Conclusion

In this work, we compared a set of sequence labeling systems. We trained and optimized every sequence labeling system for the GPRO task by means of several hyperparameter optimization algorithms and especially using the TPE. We showed that optimizing hyperparameter can be crucial. One sequence labeling system in our experiments gained an improvement of more then 60%. We showed that a naive majority vote does not bring any improvement. For this reason, we introduced and evaluated the so-called CRFVoter, a two-stage CRF tool for combining underlying sequence modeling tools (as given by the NER of our comparative study). CRFVoter gained 2% improvement compared to the best performing reference systems being examined in our study. Thus, CRFVoter may be further-developed by feeding it with the output of additional sequence labeling systems. A central theoretical outlook at this stage is to think about recursively organizing voters of the sort of CRFVoter beyond the first level by allowing different classifiers to contribute at different of these levels. In the past, such a procedure of recursive learning had been implemented by example of so-called semantic spaces [53]—see [54] for such an approach. The theoretical background is to let the system systematically abstract the results of elementary learners: As with convolutional neuronal networks, this can help to find more and more abstract, but also increasingly characteristic representations of the input data. In any event, our results and those of the other participants of BioCreative V.5 Task show that the task of recognition of genes and protein-related objects has not yet been sufficiently solved. For better recognition, a larger corpus should be generated so that the nowadays popular Deep Learning algorithms can work on this data. A kind of human-in-the-loop architecture for automatic annotation and intellectual rework would also be helpful at this point in order to successively increase and improve the amount of data.

## Abbreviations

AI: artificial intelligence
BI: biomedical imaging
BSP: biomedical signal processing
CEMP: chemical entity mention in patents
CHEMDNER: chemical compound and drug name recognition
CRF: conditional random field
F: F1-score
GM: gene mention detection
GN: gene normalization
GPRO: gene and protein related object recognition
LSTM: long short-term memory
ML: machine learning
NER: named entity recognition
P: precision
PPI: protein–protein interaction
R: recall
SMBO: sequential model-based optimization
TPE: tree-structured Parzen estimator

## Footnotes

1 http://www.uniprot.org/.

2 https://www.ncbi.nlm.nih.gov/.

3 https://www.omim.org/.

4 https://www.genecards.org/.

5 http://flybase.org/.

6 http://nlp.stanford.edu/software/CRF-NER.shtml.

7 http://cistern.cis.lmu.de/marmot/.

8 http://taku910.github.io/crfpp/.
